# Chitosan-Based Nanoparticles as Effective Drug Delivery Systems—A review

**DOI:** 10.3390/molecules28041963

**Published:** 2023-02-18

**Authors:** Karolina Jafernik, Agata Ładniak, Eliza Blicharska, Katarzyna Czarnek, Halina Ekiert, Agnieszka E. Wiącek, Agnieszka Szopa

**Affiliations:** 1 Chair and Department of Pharmaceutical Botany, Jagiellonian University, Medical College, Medyczna 9, 30-688 Kraków, Poland; 2 Department of Chemistry, Institute of Biology Sciences, Faculty of Medicine, The John Paul II Catholic University of Lublin, Konstantynów 1J, 20-708 Lublin, Poland; 3 Department of Interfacial Phenomena, Institute of Chemical Sciences, Faculty of Chemistry, Maria Curie-Skłodowska University, M. Curie-Skłodowska Sq. 3, 20-031 Lublin, Poland; 4 Department of Analytical Chemistry, Medical University of Lublin, Chodźki 4a, 20-093 Lublin, Poland; 5 Institute of Health Sciences, Faculty of Medicine, The John Paul II Catholic University of Lublin, Konstantynów 1H, 20-708 Lublin, Poland

**Keywords:** chitosan, nanoparticles, nanocomposites, nano-drugs, drug delivery efficacy

## Abstract

Chitosan-based nanoparticles (chitosan-based nanocomposites; chitosan nanoparticles; ChNPs) are promising materials that are receiving a lot of attention in the last decades. ChNPs have great potential as nanocarriers. They are able to encapsulate drugs as well as active compounds and deliver them to a specific place in the body providing a controlled release. In the article, an overview has been made of the most frequently used preparation methods, and the developed applications in medicine. The presentation of the most important information concerning ChNPs, especially chitosan’s properties in drug delivery systems (DDS), as well as the method of NPs production was quoted. Additionally, the specification and classification of the NPs’ morphological features determined their application together with the methods of attaching drugs to NPs. The latest scientific reports of the DDS using ChNPs administered orally, through the eye, on the skin and transdermally were taken into account.

## 1. Introduction

Nanomaterials have been widely used in medicine, pharmaceuticals, and cosmetics thanks to their specific features. Nanosized materials frequently have improved properties compared to the base materials they come from. In the abovementioned fields of life, the greatest interest is in natural polymers, mainly polysaccharides. Polysaccharide-based NPs are known as nontoxic, biodegradable, and physiologically stable. NPs can be involved in drug delivery systems providing controlled or sustained drug release at appropriate, therapeutic concentrations. The use of nano-size carriers may affect the improvement of different pharmaceutical processes, such as bioavailability, and pharmacokinetic profiles, as well as extend drug half-life and decrease the frequency of administration [1,2].

Among various natural polysaccharides chitosan is extensively used in health life formulations. The fact that chitosan nanoparticle (chitosan-based nanocomposites; ChNPs) properties can vary depending on the preparation methods that are used leads to applications in completely different fields. Amid naturally occurring polymers chitosan is one of the most used in biomedicine. On the commercial scale, it is derived from chitin most often by chemical deacetylation. Nevertheless, both are present in the shells of crustaceans and aquatic microorganisms (such as crabs or shrimps), fungal cell walls, and the insect exoskeleton (or wings). Structurally, chitosan is a linear polysaccharide consisting of randomly distributed D-glucosamine (deacetylated unit) and N-acetyl-D-glucosamine (acetylated unit) interspersed with a β- (1→4) bond. The molecular weight (MW) of chitosan varies from 50 to 1000 kD, with a degree of deacetylation (DD) of 30–95%, depending on the source and method of treatment. Both MW and DD determine the properties and mode of action of chitosan in biological systems [3,4,5,6,7].

This N-deacetylated chitin derivative is a valuable biopolymer for the production of nanoparticles because the functional groups present in its structure give it a unique polycationic character [8,9]. Via these reactive groups, they can be modified in a simple way by chemical activation or cross-linking; they give Ch complexing and chelating properties [8,10,11].

Chitosan is used in various industries, including pharmaceutical (as supplements acting as fat-blockers and lower cholesterol by eliminating fat and cholesterol from the body instead of allowing the body to absorb them [12]), cosmetic (where it is used in skin care products, face creams, etc. [8,13]), medical—in preparations for tissue regeneration and wound healing [8,9,14].

Among the various biopolymers available in nature, chitosan has received attention due to its inherent antimicrobial properties [9,15]. In addition, Ch is a non-toxic, biodegradable polymer that also shows high biocompatibility, a low level of immune reactions [16], and both mucoadhesive [17,18] and absorption-enhancing properties. Because of its beneficial properties, chitosan is widely used in drug delivery systems (DDS) with improved biodistribution, increased specificity, and sensitivity, and also reduced pharmacological toxicity [19,20]. Despite these attractive features, the disadvantages of Ch are its weak mechanical properties and a high rate of degradability. To overcome this problem, it is usually cross-linked and/or combined with other natural polymers or used in composites. The influence of various properties of chitosan is particularly important in relation to their usefulness in the design of innovative, multi-purpose DDS.

ChNPs have very good anticancer properties, which have been confirmed by many researchers [21,22,23,24,25,26,27,28,29,30,31,32,33,34,35,36,37,38]. They have been proven to be toxic to oral cavity [28], breast [29,30,31,32], prostate [33,34], glioblastoma [35], liver [36,37], and colon cancers [37,38], while showing satisfactory biocompatibility with normal developing cells and/or tissues. The main assessment of the anti-cancer effect of the newly formed NPs, apart from the release kinetics, was to obtain a dependence of the release on pH conditions, which is very beneficial in targeted therapy [39]. Sensitivity to pH conditions helps to overcome the problem of premature leakage of chemotherapeutic drugs during delivery. It eliminates problems related to the difficulty of the entry of drugs into cancer cells, and significant side effects in normal organs. All these reports are extremely important; however, most of them require verification in the form of in vivo studies. However, this review aims to present the latest literature reports on the effectiveness of ChNPs in relation to a specific route of administration/application of these systems with particular emphasis on oral, through the eye, on the skin, and transdermal.

## 2. Chitosan and ChNPs Utilities in Drug Delivery Systems (DDS)

### 2.1. Chemical and Biological Properties of Chitosan Used in DDS

**Figure 1:**
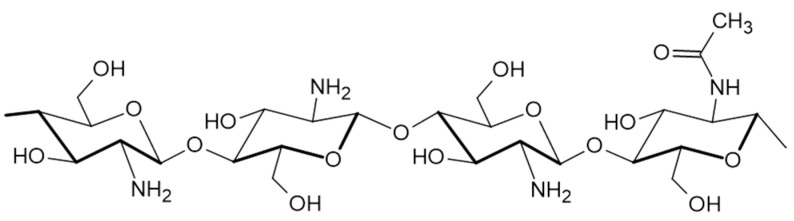
Scheme presenting the chitosan’ structure.

The most important feature of chitosan is the presence of reactive functional groups in its structure, this means amino groups at C2 and OH groups at C3 and C6 positions (Figure 1). On the one hand, the amount of amino groups reflected in DD values are inverse in proportion to degradation in the body, i.e., the higher DD (>85%) the slower the degradation. On the other hand, these groups facilitate its solubility. Abovementioned, the reactive groups also enable the chitosan chemical modification by electrostatic interactions, thus making it possible to overcome the limitations associated with the use of pure chitosan. Additionally, MW and DD are the two crucial properties of chitosan affecting the efficiency of drug encapsulation [40,41,42,43].

The unique biological and chemical chitosan properties mean that it has a well-established position in many applications in biomedical and pharmaceutical areas, primarily in the delivery of drugs or genes. The polycationic nature of chitosan renders the material to be more readily bioadhesive and soluble; it easily binds to negatively charged surfaces such as mucous membranes via electrostatic interactions. Thus, it improved the adhesion to the mucosa and extends the contact time for active substances to penetrate it. The multifarious attributes of chitosan can be exploited in anionic DDS, including small molecule drugs, as well as in polyanionic biomolecules such as nucleic acids of DNA or siRNA. Its transportability increases with charge, so it can be applied as a pH-dependent medication carrier. The chitosan penetration mechanism is also based on the interaction of the cell membrane with the positively charged polymer. Chitosan with a high deacetylation degree and high molecular weight shows the relative permeability of the epithelium, and thus can enhance the transport of polar drugs across the epithelial surfaces [5,6,7,44,45].

Chitosan is also characterized by superior biocompatibility and poor toxicity due to its natural glycosaminoglycan structural affinity. It is readily biodegradable into non-toxic metabolites that are completely absorbed by the human body. Chitosan also inhibits the efflux pump—it inhibits the transport of certain proteins on the membrane of intestinal epithelial cells or enterocytes that pump out xenobiotics, mainly drugs; these transporters are important elements in the mechanisms of drug resistance [46,47,48,49,50].

Although chitosan has many valuable functional properties, there are also a few limitations that affect its use in drug transport. It has low thermal stability and ductility and high hydrophilicity and degree of swelling. Additionally, poor solubility is an important limiting factor in its use. In fact, improving the solubility of chitosan is an essential aspect of the reasonable use of chitosan. Chemical derivatives of chitosan allow it to improve and survive the limitations. Chitosan descendants are becoming more and more popular among scientists, which means that better and better modifications are being used. The most commonly used chitosan derivatives in the pharmaceutical industry are made by acylation, carboxymethylation, quaternization, and thiolation [46,47,51].

### 2.2. ChNPs as An Improved Form of Chitosan Used in DDS

Chitosan has been considered as a potential biopolymer-based carrier that can be applied in drug delivery systems (DDS) mainly due to its biocompatibility, bioadhesive properties, and enhanced absorption effect. Chitosan-based DDS have been widely used in the form of NPs, hydrogels, and polymeric hydrogel membranes. Among these kinds of chitosan-based DDS, NPs are the most promising formulations for pharmaceutical use. Chitosan nanoparticles (ChNPs) exhibit the same attributes as natural or chemically modified polymers. The preparation of ChNPs takes place under mild conditions due to the chemical properties of chitosan—it is soluble in acidic aqueous solutions at room temperature, and no toxic organic solvents or heat are required.

A wide range of drugs can be incorporated into ChNPsDDS, including small molecules, proteins, and polynucleotides. The nanoparticles can release the encapsulated drug in a controlled manner. ChNPs are of great interest to researchers around the world. More than 1500 papers describing and examining the mechanism of ChNP activity have already been produced. In fact, ChNPs can be used in every possible way of drug delivery—oral, ocular, nasal, pulmonary, buccal, periodontal, vaginal, cutaneous and transdermal drug delivery and wound healing, but also in the delivery of vaccines and genes [52,53,54,55].

## 3. General Characterization of NPs of DDS Application

### 3.1. Morphological Features of NPs Influencing Their Application

The basic definition of NPs in terms of use in nanomedicine is as follows: colloidal medicine carrier systems that consist of native or artificial polymers, typically in the size range of 10 to 100 nm. In this case, particle size is actually the key factor in determining the absorption, distribution, accumulation and eradication of NPs in the human body [51,55,56]. The particle size can influence the NPs internalization (in the endocytotic pathway) and thus can determine the capacity and the transmembrane transport rate of NPs. The uptake of NPs by intestinal cells or M cells after oral administration is size-dependent, which affects their efficiency and oral absorption rate. Small particles are absorbed by the intestinal cells, while large particles are mainly absorbed by M cells, the spleen and renal excretion [51,56]. In general, NPs larger than 10 nm are able to avoid renal clearance and penetrate into tissues. Although 10–20 nm particles can be widely distributed in various organs through tight endothelial junctions, they will also be quickly excreted through the glomeruli. NPs larger than 200 nm can be absorbed quickly by the mononuclear phagocytic system and concentrated in the liver and spleen. In conclusion, in most cases, small-size NPs are advantageous for long circulation and distribution in diverse tissues. However, it should be borne in mind that the particle size should not be as small as possible, because too small particles will lead to increased instability of the nanosystem under physiological conditions and will not bring the expected effect [57].

Another fundamental aspect is the shape of the NPs, which influences and determines their biological mechanisms in vivo. Phagocytosis is the first hindrance that NPs encounter in the blood system; therefore, macrophage phagocytosis is strongly shape-dependent. The form of NPs can affect their adhesion and internalization. In addition to the effect on blood circulation time, in some cases, the shape of the particles can also influence their allocation in vivo. Non-spherical NPs (ellipse, bar, cylinder, star, ring, and other arduous shapes) can have a great effect on the transmission in the vascular system and their distribution. Although most currently studied NPs are spherical in shape, the unique attributes of non-spherical NPs can have a significant impact on circulation and transfer [58].

Surface charge and hydrophobicity are also important NPs parameters influencing their biological courses in vivo. Hydrophilic nanomaterials can reduce the reticular endothelial system response, while hydrophobic ones can improve the NPs penetration. Therefore, it seems understandable to combine the two types of materials in the NPs design in a defined ratio to allow the best in vivo endurance and most favorable targeting to disordered sites. The surface charge may influence the adsorption of some specific structures, thus causing the recognition, phagocyting, and elimination of NPs by macrophages, which may next affect their carrying and assignment in the organism [5,47,59].

In nanomedicine, targeting nanocarriers reflects an increased concentration of particles at a disordered place. Compared to passive targeting (size, shape, surface charge, and hydrophobicity) of NPs, active targeting (modified by ligands) can more actively and efficiently transport NPs to the target site. In addition, the modification of the target ligands may also reduce the clearance of NPs. Appropriate NPs modification with ligands based on the features of the disease site is considered one of the future research directions of nanocarrier targeting studies [60,61,62].

### 3.2. Classification of NPs According to Their Morphological Features

NPs are often divided into two groups based on their structure: nanospheres and nanocapsules. The nanospheres are characterized by a solid structure with a homogeneous matrix structure in which the resources are evenly distributed, while nanocapsules form the traditional hollow shell structure consisting of a polymer membrane and an inner core containing the drug. Both materials are the main target for the incorporation of active compounds, have a high load-bearing capacity, and provide controlled delivery [61,62].

Modern nano-drugs are more successful than current drugs or drugs combined with microstructures due to their capability to target at the right site, constant and controlled release, improved absorption rate and bioavailability, and enhanced stability of the therapeutic factors. The NPs size, surface charge, surface modification, hydrophobicity, and high surface-to-volume ratio are aspects that control the targeting capabilities of the nano-drugs. These properties are also very important in reducing the drug dose and the frequency of dosage, thereby decreasing the toxicity and possible aftereffects of chemotherapeutics [61,62,63].

## 4. Methods of Attaching Drugs to NPs

Chemotherapeutics can easily bind to nanocarriers due to covalent bonding or adsorption. Two methods of drug loading on NPs are used: when obtaining particles (incorporation process), and after particle formation (incubation process).

The drug is physically fixed in the matrix or absorbed on the surface. Charging efficiency largely depends on the chosen method of nanoparticle preparation and the physicochemical properties of the drug. Although maximum loading can be achieved by including the drug during particle formation, it can be influenced by among others type of the preparation method or the presence of other additives [64,65].

Chitosan-based polymer NPs act as excellent drug shippers due to some of the inherent beneficial properties of chitosan—ease of preparation, non-toxic character, bioactivity, biocompatibility, biodegradability, as well as cationic character. ChNPs good mucoadhesive capacity improved bioavailability and the dissolution rate of hydrophobic drugs. In addition, chitosan enhances the stability of unstable drugs, their bioavailability, and controlled drug release because the molecules are outside the nanoscale range to effectively overcome barriers and increase permeability. Both water-soluble and water-insoluble drugs can be incorporated into ChNPs. Water-soluble drugs are mixed with the chitosan solution to form a homogeneous mixture, and then the particles can be prepared by any method. Water-insoluble drugs and drugs that can precipitate in solutions at an acidic pH can be charged after particle formation by soaking the preformed NPs in a saturated drug solution [64,65,66].

The release of a drug from ChNPs depends on the morphology, size, and density of the particle system and the degree of cross-linking, the physicochemical properties of the medicines, as well as the adjuvants used. In vitro delivery is also dependent on pH, polarity, and the presence of enzymes in the delivery medium. In most cases, drug release follows more than one type of mechanism. In the case of drugs adsorbed on the surface of ChNPs, as well as drugs enclosed in the surface layer of NPs, the medicine immediately dissolves after contact with the release medium. This type of drug delivery leads to a burst effect in the early stages of dissolution. Otherwise, the release of the drug by diffusion is at the beginning slow and then it gets faster [64].

The most common ChNP shapes are nanospheres, nanocapsules, and nanofibers, which are detailed in Table 1.

## 5. Methods of Producing ChNPs

Several types of NP production methods are known such as physicochemical processes (NPs are formed after precipitation, with the use of preformed polymers, whereas the yield is controlled by the rate of both evaporation, diffusion, or inverse salting out of the emulsifier-solvent); chemical synthesis of macromolecules (polymerization or interfacial polycondensation in situ), and mechanical processes (NPs are produced by both high-pressure homogenization, under ultrasound as well during high energy wet milling).

**Figure 2:**
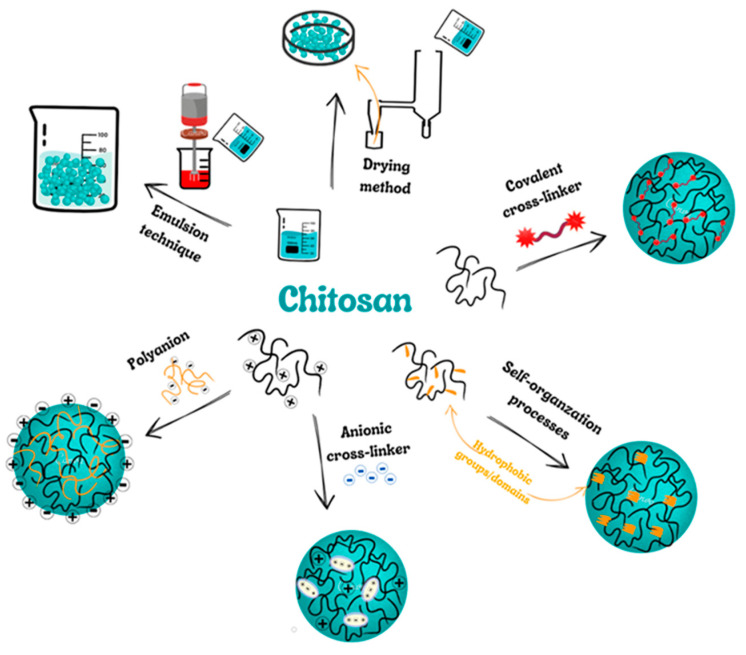
Scheme presenting the most popular methods of chitosan nanoparticles (ChNPs) producing.

The methods that are mainly used to produce ChNPs are ion gelation, self-assembly, emulsification and cross-linking, complexation with polyelectrolytes, and drying processes (Figure 2) [64,65,66,67].

### 5.1. Covalent Cross-Linking

This method focuses on the coupling of two or more molecules to each other through covalent chemical bonds. In the ChNPs instance, mentioned bonds are created between chitosan (or derivatives of Ch) and the cross-linker. Crosslinking agents most often have multiple functional groups responsible for covalent crosslinking. Overall, thanks to bridging links between individual polymer chains, nanomaterials are able to make a three-dimensional structure (3D). The most popular cross-linking agents in this field are glutaraldehyde, genipin as well polyethylene glycol [64].

### 5.2. Self-Organization Processes

Self-organization relies on the joining of molecules, macromolecules, or composites together to organize a 3D network or another structure having new, distinctive properties. That process can happen at the molecular or supramolecular stage. It can arise through self-connection or connection with further compounds as a result of hydrogen bonding, van der Waals forces, and ionic or hydrophobic interactions. It may also be due to an inclusion/complexation mechanism such as an iodine-starch inclusion complex. The aqueous environment of amphiphilic derivatives of polymers favors the self-assembling of NPs. The hydrophobic interactions between the amphiphilic polymer components contribute to minimizing the interfacial energy allowing the NPs creation. Self-organizing ChNPs are especially useful for encapsulating both hydrophilic and lipophilic drugs [65].

### 5.3. Ion Cross-Linking (Ion Gelation)

Ion cross-linking is one of the widespread methods for producing ChNPs, especially for loading biopharmaceuticals. The main benefit of physical chitosan cross-linking is associated with the mild conditions achieved. This method does not require the application of organic solvents, high temperatures as well vigorous mixing. Therefore, it can retain bioactive molecules such as proteins, and DNA. Two main groups of anionic crosslinkers have been used: low MW anionic molecules such as cyclodextrin or tripolyphosphate (TPP) derivatives, and negatively-charged macromolecules among which can be distinguished poly-y-glutamic acid, dextran sulfate, hyaluronic acid and sodium alginate [65].

### 5.4. Polyelectrolyte Complex (PEC)

The method of production of self-organizing ChNPs is to form polyelectrolyte complexes (PECs) with polyanions. PECs are formed in a solution of two different polyelectrolytes, i.e., polycation (having the positive charge) and polyanion (of negative charge) or their respective salts. PEC creation is possible due to strong Coulombic interactions between oppositely charged polyelectrolytes. As a result, the least partial charge neutralization of the polymers occurs. Moreover, inter-macromolecular interactions (such as Van der Waals forces), hydrogen bonding, and hydrophobic interactions are affected in the PEC structure formation [64,65].

Creation of the PEC usually involves two or three steps. The first stage is prompt and brings on the formation of an accidental primary complex with meaningful distortions in the configuration of the polymer chains. The secondary complex is then made by rearranging the existent bonds within the complexes. That step involves the formation of new bonds, e.g., hydrogen, electrostatic, and hydrophobic interactions. Finally, under certain conditions, primary and secondary complexes can aggregate (presumably through hydrophobic interactions) leading to the creation of varied stable structures, e.g., filaments, entangled aggregates, and ordered networks. The production of PEC with chitosan is a safe process (without organic solvents, chemical cross-linkers, and surfactants) [65,66].

### 5.5. Emulsion Technique

In general, the particles formed in the emulsion system have a nanocapsule structure, high drug-loading efficiency, and good bioavailability. NPs can be obtained in various ways using the nanoemulsification technique. Some are low-power emulsification methods, but such processes usually require high mechanical energy to obtain small droplet sizes. The most common method used to produce ChNPs is water-in-oil emulsification in which an aqueous chitosan solution is emulsified in the oil phase. Surfactants are used to stabilize the NPs [64,65].

### 5.6. Drying Methods

In the drying process, water or solvents are divided by evaporation from liquids, solids, or semi-solids. Hot air, spray drying, microwave oven, freezing, supercritical drying, and open-air drying are popular drying methods. Spray and supercritical drying techniques are the most often used in ChNP production because they are simple, fast, continuous, repeatable, and easy to adapt to the active material. They are reduced in one phase with no modifications. These methods are important to obtain different particle sizes and high stability [64,65].

In summary, the main advantages and disadvantages of the ChNPs production methods are presented in Table 2.

## 6. Methods of DDS through ChNPs

ChNPs are widely used in the fields of medicine, biology, and pharmacy, in particular, they are valued as drug-delivery vehicles. The main features of ChNPs that are used in drug delivery are the ability to control drug release, reduce doses and thus side effects, improve drug stability, and thus increase efficacy and bioavailability.

**Figure 3:**
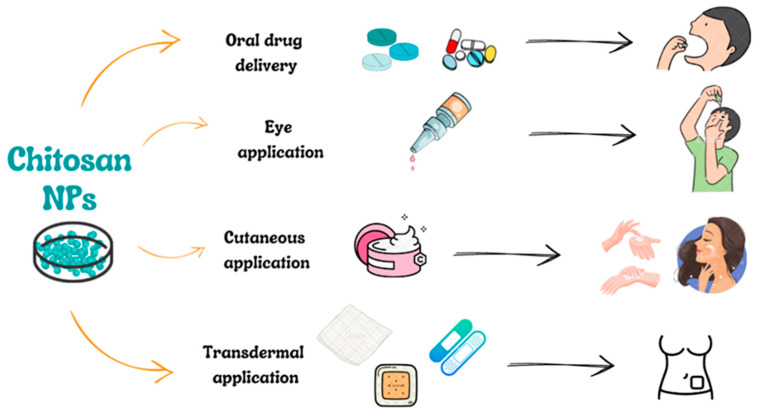
Scheme presenting the most popular ways of chitosan nanoparticles (ChNPs) applications.

Most often, ChNPs as drug carriers are used orally, through the eye, on the skin, and transdermally (Figure 3).

### 6.1. Oral Drug Delivery

Oral administration is the most common and simplest method of drug delivery. An important aspect is also that it is a non-invasive method. On the other hand, this is a slow process, and some medications can be adversely affected by body fluids, such as stomach acid. The ideal carrier for drugs should be a structure that forms stable complexes with active substances in the gastrointestinal tract while protecting them against degradation by delivering them to target cells. The carrier also requires non-toxicity, biodegradability, and biocompatibility—in the case of ChNPs these criteria are met. The following is an overview of the most recent oral drug delivery research using ChNPs as a drug carrier.

Saraswati et al. described the preparation of oral administration of complexes containing 10% Channa striata protein hydrolysate (snake fish), chitosan-PEG 4000, and chitosan-PEG 6000 NPs. The developed nanocomplexes were aimed at lowering the sugar level in diabetic rats. The experiment was performed on male Sprague-Dawley rats. Diabetes was initiated by one injection of streptozotocin (1 mL) in each formulation. It was shown that the preparation containing 10% Channa striata protein hydrolysate and PEG6000 was the most efficient in blood glucose level lowering. Glucose concentration has also been shown to decrease after daily oral chitosan-PEG 4000 NPs for 21 days of administration. Plasma concentrations of cholesterol, triglycerides, LDL, and HDL in diabetic rats, were also lower in treated than in untreated animals [77].

Abdel-Moneim et al. also focused on anti-diabetes treatments. They developed a new formula for the oral administration of ChNPs with polytadine, which improved the therapeutic potential of polytadine. In vivo studies showed that the developed complex showed significant anti-diabetic efficacy in diabetic rats compared to polytadine alone, which indicates that ChNPs are promising carriers for polytadine for the treatment of type 2 diabetes [78].

Mohanbhai and his team focused on developing a formulation that can be used in Crohn’s disease (CD) and ulcerative colitis (UC). Melatonin has proven to be a powerful anti-inflammatory agent for the treatment of UC. The problem, however, was the poor solubility that limits its therapeutic potential. Scientists developed ChNPs coated with Eudragit-S-100 loaded with melatonin and demonstrated anti-inflammatory efficacy in terms of NO scavenging activity in LPS-challenged macrophages in vitro. In addition, the colon-targeted oral chitosan nanopreparation showed remarkable protection in the in vivo UC mouse model by improving the overall pathological parameters, histo-architectural protection, goblet cell depletion, and immune cell infiltration, which can be extrapolated to clinical trials [79].

Li et al. focused on designing NPs that could be used in spinal cord injury (SCI). The result was chitosan-modified hollow manganese dioxide NPs (ChNPs/MgO_2_), which were designed to transport resveratrol and facilitate its passage through the blood-spinal barrier (BSCB). Resveratrol (Res), a poorly soluble compound, was adsorbed into NPs ChNPs/MgO_2_ at a particle size of about 130 nm, and the Res loading was equal to 21.39%. In an in vitro dissolution experiment, the release of Res from the loaded sample (CMR) showed a slow release behavior and was about 87% after 36 h. Under in vitro and in vivo tests it was shown that CMR can significantly alleviate oxidative stress by lowering reactive oxygen species (ROS), superoxide dismutase (SOD), malondialdehyde (MDA), and increasing glutathione peroxidase (GSH) levels. In addition, in immunofluorescence (iNOS, IL-1β and Cl caspase-3) and Western blots (COX-2, iNOS, IL-10, IL-1β, Cl caspase-3, Bax and Bcl-2), the expression of related factors were used for detection, which confirmed that CMR can also reduce neurons inflammation and apoptosis. These studies indicate that the use of ChNPs, as DDS, is promising for SCI treatment [59].

ChNPs also play an important role as carriers in anti-cancer drugs. One example is methotrexate, widely used as a chemotherapeutic agent. Researchers led by Coutinho developed NPs containing fucoidan and chitosan that were loaded with methotrexate for the treatment of lung cancer. The obtained results showed that methotrexate-loaded NPs were seven times more effective in inhibiting the proliferation of lung cancer A549 cells than the drug administered alone [80].

Xavier et al. focused on improving the oral administration of palitaxel (PTX). They developed nanocapsules based on polo (butyl cyanoacrylate) by interfacial polymerization of a surface coated with chitosan. The resulting nanocapsules were loaded with PTX by adding copaiba oil to the inside. Subsequently, the nanocapsules were tested for oral administration. The results showed good mucoadhesive capacities and good association (9%) with the mucosa of the rats tested [81].

Abdulmalek et al. tested a combination of ChNPs with bee venom and the result of this complex on hepatocellular carcinoma (HCC). Targeted bee venom ChNPs were found to have higher cytotoxicity to HepG2 than SMMC-7721 cells, as well as the highest cellular uptake and a high reduction in cell migration leading to better cancer inhibition. It also facilitated cancer cell death in EGFR overexpressing HepG2 cells by stimulating reactive oxygen species and mitochondrial-dependent pathways, inhibiting the EGFR-stimulated MEK/ERK pathway, and increasing p38-MAPK levels compared to native bee venom. In mice with hepatocellular carcinoma (HCC), it has an anticancer influence. It also improved the function and architecture of the liver without noticeable toxic adverse effects as well as lowering cancer growth by induction of apoptosis [82].

Sharma et al. developed a ChNPs loaded with Carvedilol (beta-blocker), a drug with limited effects due to its hydrophobic nature. The NPs were created with the ion gelling technique. The results of pharmacokinetic tests indicate that the resulting NPs have a much higher bioavailability than the free drug, which suggests the possibility of ChNP use as a carrier for poorly water-soluble drugs [83].

ChNPs can also be used as vehicles for the intravenous distribution of polysaccharides, such as those of Ophiopogon japonicus (OJP). OJP is used to treat abnormalities in the intestinal barrier, including inflammatory bowel disorder; its absorption is low with oral administration. Lin et al. developed NPs containing OJP/chitosan and whey protein. The research shows that the resulting nanocomplexes had a positive effect on the integrity of the intestinal epithelium barrier and protected it against the damage caused by inflammation caused by lipopolysaccharide [84].

### 6.2. Eye Application

Hassan et al. designed an eye drop formulation with ChNPs to treat fungal keratitis. The formulation was prepared using the high-pressure homogenization technique and was characterized for various parameters to check their suitability. The optimized formulation successfully produced irregular spherical particles up to 200 nm in size and a polydispersity index (PDI) below 0.2 nm. The optimized formula also showed a high mucoadhesive capacity, which suggests greater retention on the eye mucosa with low eye irritation, as highlighted by the HET-CAM test (hen egg allantoic membrane). An in vitro drug release study through the dialysis membrane showed both a diffusion and a swelling-controlled release pattern for the optimized formulation. An ex-vivo corneal permeation study on goat corneal tissues using the Franz diffusion cell also showed a steady increase in drug permeation over time. In addition, the optimized formulation proved to be non-irritating and safe for the eyes in ex-vivo transcorneal toxicity studies on goat corneal tissues. The results indicate that the design of the proposed nanometric formulation is a promising step toward treating external eye diseases with positive features such as high patient compliance, controlled drug delivery, extended residence time of the drug in front of the cornea, and increased eye bioavailability [85].

Another problem with ophthalmic drug delivery is delivery to the posterior segment of the eye. One of the most commonly used steroids for inflammation is dexamethasone (DEX). Yun et al. started to develop complexes containing DEX-glycol and chitosan. The designed nanopreparation was tested on Japanese white rabbits to check whether the NPs cause eye irritation. The obtained results indicated that there was no conjunctival hyperemia, corneal opacity, or iris inflammatory exudate. Fluorescein staining tests showed no ulceration or defects in the corneal epithelial layer. The proposed NPs have good ocular tolerance and showed a longer time on the cornea than DEX in an aqueous solution [86].

Rathore et al. conducted research aimed at the formulation of ChNPs loaded with insulin in order to improve the systemic absorption of insulin by the eye, which would have the effect of reducing the administration of painful and unpleasant insulin injections in people with insulin-dependent diabetes. The manufactured NPs (C4T4I4) were characterized by a positive charge, a particle size of 215 ± 2.5 nm, and an entrapment efficiency of 65.89 ± 4.3%. In vitro drug release showed a sustained release of insulin, with 77.2% release observed after 12 h, leading to the assumption of a non-vigorous diffusion release mechanism. The carried study exposed that the insulin-loaded NPs had good mucoadhesive properties and better permeation properties than free insulin. Additionally, the prepared NPs showed adequate stability (determined by particle size stability) within six months of storage. The NPs were found to be non-irritating to eye tissues and showed a significant reduction in blood glucose levels in vivo. The results of this study suggest that the chitosan nanoparticle system may act as an important carrier system for insulin with increased stability and efficacy [87].

### 6.3. Cutaneous and Transdermal Application

Thanks to their unique properties, ChNPs are also attractive for biomedical applications, such as the cutaneous and transdermal skin delivery systems of cosmetic components and drugs, which increasingly constitute an alternative to oral route hindrances. Ta et al. proved that ChNPs were not toxic to human skin fibroblasts. Studies have also shown their ability to penetrate pig skin and accumulate in the dermis layer. ChNPs of three different chitosan molecular weights, low-medium-high (LMW, MMW, HMW—respectively) were prepared by the ionic gelation process, using two kinds of crosslinkers, sodium tripolyphosphate (TPP) and Acacia; they were a smooth, positive charged and spherical shape with a size of 200–300 nm, as well were uniformly distributed. Additionally, they showed that the NP particle sizes were strictly dependent on the chitosan mass but also the concentration of both Ch and the crosslinkers. Based on this they suggested that ChNPs can be ideal candidates in the cosmetic industry and in drug delivery systems by the skin [88].

In turn, Abdel-Hafez et al. [89], based on the carried out studies of ex vivo permeation, proved that even smaller ChNPs in the range of 33.85–199.23 nm (curcumin-loaded) were able to skin penetrate mainly the appendageal route. Curcumin was chosen because it is a therapeutic agent with the possibility of many different applications. For example, it has proven effectiveness in diffusing from collagen-chitosan scaffolds, supporting wound healing [90]. Abdel-Hafez and co-workers demonstrated that manufactured NPs were found in the hair follicles, from which curcumin moves into the deeper skin layers and other directions [89].

The effectiveness of the transdermal delivery-loaded curcumin chitosan carriers was also confirmed by Truong’ research team. They prepared the chitosan-coated nanostructured lipid carriers (ChNLCs) with encapsulated tetrahydrocurcumin (THC) as a successful agent for breast cancer treatment. Prepared by high-shear homogenization, ChNLCs-THC were negatively charged particles with a size of 244 ± 18 nm. Additionally, studies showed that carriers enabled the sustained release of THC at pHs 7.4 and 5.5. Fabricated ChNLCs-THC demonstrated remarkably enhanced skin permeation as well as cytotoxicity towards cells (breast cancer) than the unencapsulated THC [91].

In contrast, Talib et al., using chitosan and chondroitin sulfate, prepared NPs using the ionic gelation method which next were loaded in a transdermal patch. They were stable within 6 months and the patch contained uniformly distributed, positively charged, spherical NPs of the size of 234 nm with encapsulated artemether. Release and ex vivo tests revealed the uniform release of drugs from NPs via a diffusion-controlled mechanism, which was additionally enhanced by the presence of olive oil (as a permeation enhancer) in the patch [92].

The strengthened targeting potential of the NPs in the oily conditions was also observed by Tolentino and co-workers. They developed chitosan(ChNPs) and hyaluronic acid-based NPs (HyalNPs) with entrapped clindamycin as a potentially effective drug delivery system in acne treatment. Similar sizes (362 ± 19 nm and 417 ± 9 nm, Ch- and Hyal-, respectively) of NPs, despite opposite charges, both demonstrated enhanced targeted clindamycin-delivery into the pilosebaceous arrangement compared to the commercially available preparations (not containing Ch and Hyal) [93].

Remarkably, drug or active substance delivery to the pilosebaceous arrangement may also reinforce or exacerbate further by the use of iontophoresis (IP) [94]. Such an observation was made by the Takeuchi research team, who investigated the possibility of using poly(dl-lactide-co-glycolide) NPs (98.4 ± 36.8 nm) coated with chitosan hydroxypropyltrimonium chloride in allergen immunotherapy on the skin. Such in vivo immunoreactivity tests proved that model antigen (egg-white lysozyme (HEL)) entrapped in NPs, was successfully delivered to the hair follicles. What is more, transdermal application was more effective than subcutaneous injection [94].

Recent available literature reports that chitosan-based NPs (ChNPs) are able to promote antimicrobial and anti-inflammatory activity in targeted therapy for cutaneous pathogens [95] as well as wound healing [90,96,97]. Chitosan possesses excellent antimicrobial properties [15,98,99]. This encouraged the researchers Friedman et al. to fabricate the chitosan–alginate NPs for targeted therapy for different pathogens (e.g., cutaneous) with particular emphasis on the pathogenesis of acne.

Chitosan–alginate NPs have been proven to be effective antimicrobial and anti-inflammatory agents, demonstrating direct in vitro activity against bacteria such as Propionibacterium acnes, inducing the disruption of the cell membrane and inhibiting P. acnes which is responsible for the production of inflammatory cytokine in human monocytes and/or keratinocytes. Moreover, they turned out to be effective carriers for antiacne drugs, i.e., benzoyl peroxide (BP). BP entrapped in chitosan–alginate NPs were more effective against P. acnes and biocompatible with eukaryotic cells than BP alone [95].

The research team of Lopes Rocha Correa manufactured the melatonin-loaded lecithin-ChNPs (MEL-NPs) that induced fibroblast proliferation, angiogenesis, increased collagen deposition, and finally accelerated wound closure faster in diabetic rats. The prepared MEL-NPs are positively charged and have a relatively small size of 160 nm. Carried experiments in vivo proved that MEL-NP is efficient in improving wound healing in the rat model even for those with diabetes mellitus syndrome [96]. A similar observation was provided by Choudhary et al. who investigated quercetin-loaded chitosan tripolyphosphate NPs (QChNPs) with a size of 361.16 ± 9.72 nm. In vivo tests in Wistar rats revealed that the most effective wound healing treatment seems to be NPs consisting of 0.03% of quercetin. The examined QChNPs influenced cytokine behavior and growth factors of inflammatory and/or proliferative processes thus accelerating the course of wound healing [97].

## 7. Conclusions

Among the available natural polymers, chitosan (Ch) and especially its nanoparticles (ChNPs) seem to be the most attractive to be used for controlled release in different DDS. Ch has been strongly indicated as a suitable functional material in view of its excellent biocompatibility, biodegradability, and adsorption properties. This interest is partly attributed to its structure abundant in functional amino and hydroxyl groups. These moieties enable chitosan modifications in a simple way, as well as effectively binding active substances, most importantly for the precisely dosed and sustained release of bioactive substances or drugs. Moreover, ChNPs have gained more attention due to their better stability, low toxicity, and simple and mild preparation method; providing versatile routes of administration. The presented review was focused on the significant role of ChNPs in functional DDS. It brings knowledge of the preparation, different forms, and application of ChNPs, and gives short overviews of recent developments in the main fields of drug delivery, i.e., with different administration routes including oral, eye, cutaneous as well transdermal.

## Figures and Tables

**Table 1:** Characteristics of the most commonly used shapes of chitosan nanoparticles (ChNPs).

Shapes of Structures	Chemical Construction	Figure	Application
**Nanospheres**	- matrix system, - the drug is most often absorbed on the surface or enclosed in a molecule	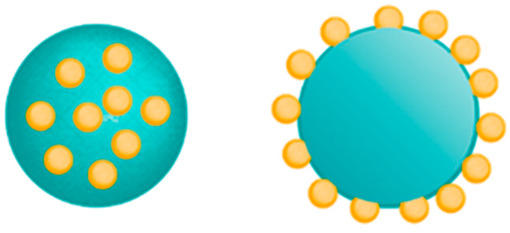	- prolonged and sustainable drug release, - periodontal and osseous repair, - increase the efficiency of the photothermal conversion, - use in chemotherapy drugs
**Nanocapsules**	- hollow-core structures, - the drug is generally included in an oily core covered with a chitosan coating	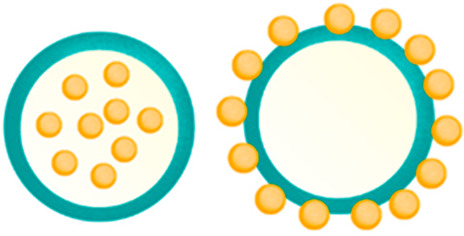	- sequential drug release, - used in cancer therapy, reduce the viability of cancer cells, - promoting wound healing
**Nanofibers**	- specific structure with of -NH_2_ and -OH groups along its shape, - produced by electrospinning from polymers solution, - drugs are loaded during fibers production or mixed with finished fibers	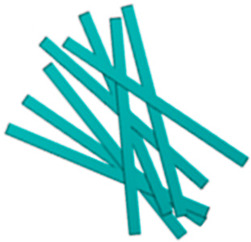	- long drug release, delivery of drugs, peptides, and vaccine antigens, - bone regeneration, - smooth muscle tissue engineering, - scaffolds for dermal wound healing

**Table 2:** Advantages and disadvantages of the ChNPs production methods [20,64,65,68,69,70,71,72,73,74,75,76].

Type of Method	Advantages	Disadvantages
**Covalent cross-** **linking**	- possible to obtain small-size NPs with a narrow distribution [68] - high mechanical properties of NPs [69]	- cross-linking agent causes overt toxicity and drug integrity issues [68], - absence of pH-dependence drug release [69], - lack of swelling [70]
**Self-organization processes** **and** **Polyelectrolyte complex (PEC)**	- simple and mild preparation method [68,71], - without harsh conditions [71], - eliminates toxic substances [71], - may form complexes with natural anionic materials [68], - especially favorable for encapsulating hydrophilic and lipophilic drugs [68], - protect the loaded active compound from adverse external conditions and control its release [72,73]	- requires constant control of pH, because may occur tend to aggregate when PEC particles are formed [69,74], - the mechanical properties and permeability of the products are strongly influenced by the properties of the starting material, mixing ratio, mixing order and conditions of reaction [69,74]
**Ion cross-linking (ion gelation)**	- straightforward technique [68,71], - without any harmful crosslinker or solvents [68,71], - reduces undesirable effects [68,71], - improves biocompatibility [68,71], - gives the particle size control based on the chitosan -to-stabilizer ratio [68,71]	- poor bioavailability upon oral administration [75], - can affect intrinsic properties of chitosan [65], - poor stability [20], - encapsulation efficiency is relatively lower than other methods [20]
**Emulsion** **technique**	- better particle size control [71], - appropriate for both hydrophobic and hydrophilic drugs [71]	- require strong cross-linking agents [71], - require high mechanical energy to obtain small size of droplets [71], - difficulties with the elimination of the residual cross-linking agents [71]
**Drying methods**	- simple and fast method [76], - reducing particle size and creating narrow particle distribution [76], - have better quality in terms of its low moisture content and flowability which offers greater shelf life to the product [76]	- various process parameters should be controlled to obtain the desired size of particles [64], - sometimes requires the cross-linking agent, causing a process to be multistep [64], - high temperature during the process could cause the degradation of sensitive compounds [76]

## Data Availability

Not applicable.

## References

[1] Jurak M., Wiącek A.E., Ładniak A., Przykaza K., Szafran K. (2021). What Affects the Biocompatibility of Polymers?. Adv. Colloid Interface Sci..

[2] Kim H.S., Sun X., Lee J.H., Kim H.W., Fu X., Leong K.W. (2019). Advanced Drug Delivery Systems and Artificial Skin Grafts for Skin Wound Healing. Adv. Drug Deliv. Rev..

[3] Upadhyaya L., Singh J., Agarwal V., Pandey A.C., Verma S.P., Das P., Tewari R.P. (2015). Efficient Water Soluble Nanostructured ZnO Grafted O-Carboxymethyl Chitosan/Curcumin-Nanocomposite for Cancer Therapy. Process. Biochem..

[4] Varum K., Ottoy M., Smidsrod O. (2001). Acid Hydrolysis of Chitosans. Carbohydr. Polym..

[5] Kumar A., Vimal A., Kumar A. (2016). Why Chitosan? From Properties to Perspective of Mucosal Drug Delivery. Int. J. Biol. Macromol..

[6] Elgadir M.A., Uddin M.S., Ferdosh S., Adam A., Chowdhury A.J.K., Sarker M.Z.I. (2015). Impact of Chitosan Composites and Chitosan Nanoparticle Composites on Various Drug Delivery Systems: A Review. J. Food Drug Anal..

[7] Lim C., Hwang D.S., Lee D.W. (2021). Intermolecular Interactions of Chitosan: Degree of Acetylation and Molecular Weight. Carbohydr. Polym..

[8] Jiménez-Gómez C.P., Cecilia J.A. (2020). Chitosan: A Natural Biopolymer with a Wide and Varied Range of Applications. Molecules.

[9] Song E.-H., Shang J., Ratner D.M. (2012). Polysaccharides. Polymer Science: A Comprehensive Reference.

[10] Qiao F., Ke J., Liu Y., Pei B., Hu Q., Tang B.Z., Wang Z. (2020). Cationic Quaternized Chitosan Bioconjugates with Aggregation-Induced Emission Features for Cell Imaging. Carbohydr. Polym..

[11] Ma J., Wang Y., Lu R. (2022). Mechanism and Application of Chitosan and Its Derivatives in Promoting Permeation in Transdermal Drug Delivery Systems: A Review. Pharmaceuticals.

[12] Moraru C., Mincea M.M., Frandes M., Timar B., Ostafe V. (2018). A Meta-Analysis on Randomised Controlled Clinical Trials Evaluating the Effect of the Dietary Supplement Chitosan on Weight Loss, Lipid Parameters and Blood Pressure. Medicina.

[13] Guzmán E., Ortega F., Rubio R.G. (2022). Chitosan: A Promising Multifunctional Cosmetic Ingredient for Skin and Hair Care. Cosmetics.

[14] Pramanik S., Sali V. (2021). Connecting the Dots in Drug Delivery: A Tour d’horizon of Chitosan-Based Nanocarriers System. Int. J. Biol. Macromol..

[15] Ładniak A., Jurak M., Palusińska-Szysz M., Wiącek A.E. (2022). The Influence of Polysaccharides/TiO_2_ on the Model Membranes of Dipalmitoylphosphatidylglycerol and Bacterial Lipids. Molecules.

[16] Dimassi S., Tabary N., Chai F., Blanchemain N., Martel B. (2018). Sulfonated and Sulfated Chitosan Derivatives for Biomedical Applications: A Review. Carbohydr. Polym..

[17] Ways T.M.M., Lau W.M., Khutoryanskiy V.V. (2018). Chitosan and Its Derivatives for Application in Mucoadhesive Drug Delivery Systems. Polymers.

[18] Hasnain M.S., Nayak A.K. (2022). Chitosan as Mucoadhesive Polymer in Drug Delivery. Chitosan in Drug Delivery.

[19] Elkomy M.H., Ali A.A., Eid H.M. (2022). Chitosan on the Surface of Nanoparticles for Enhanced Drug Delivery: A Comprehensive Review. J. Control. Release.

[20] Cao S., Deng Y., Zhang L., Aleahmad M. (2022). Chitosan Nanoparticles, as Biological Macromolecule-Based Drug Delivery Systems to Improve the Healing Potential of Artificial Neural Guidance Channels: A Review. Int. J. Biol. Macromol..

[21] Tousian B., Ghasemi M.H., Khosravi A.R. (2022). Targeted Chitosan Nanoparticles Embedded into Graphene Oxide Functionalized with Caffeic Acid as a Potential Drug Delivery System: New Insight into Cancer Therapy. Int. J. Biol. Macromol..

[22] Mi Y., Chen Y., Gu G., Miao Q., Tan W., Li Q., Guo Z. (2021). New Synthetic Adriamycin-Incorporated Chitosan Nanoparticles with Enhanced Antioxidant, Antitumor Activities and pH-Sensitive Drug Release. Carbohydr. Polym..

[23] Wang F., Li J., Tang X., Huang K., Chen L. (2020). Polyelectrolyte Three Layer Nanoparticles of Chitosan/Dextran Sulfate/Chitosan for Dual Drug Delivery. Colloids Surf. B Biointerfaces.

[24] Rajalakshmi R., Sivaselvam S., Ponpandian N. (2021). Chitosan Grafted Fe-Doped WO_3_ Decorated with Gold Nanoparticles for Stimuli-Responsive Drug Delivery Systems. Mater. Lett..

[25] Sohail R., Abbas S.R. (2020). Evaluation of Amygdalin-Loaded Alginate-Chitosan Nanoparticles as Biocompatible Drug Delivery Carriers for Anticancerous Efficacy. Int. J. Biol. Macromol..

[26] Chen X., Bremner D.H., Ye Y., Lou J., Niu S., Zhu L.M. (2021). A Dual-Prodrug Nanoparticle Based on Chitosan Oligosaccharide for Enhanced Tumor-Targeted Drug Delivery. Colloids Surf. A Physicochem. Eng. Asp..

[27] Cardoso V.M.d.O., Brito N.A.P.d., Ferreira N.N., Boni F.I., Ferreira L.M.B., Carvalho S.G., Gremião M.P.D. (2021). Design of Mucoadhesive Gellan Gum and Chitosan Nanoparticles Intended for Colon-Specific Delivery of Peptide Drugs. Colloids Surf. A Physicochem. Eng. Asp..

[28] Matos B.N., Pereira M.N., Bravo M.d.O., Cunha-Filho M., Saldanha-Araújo F., Gratieri T., Gelfuso G.M. (2020). Chitosan Nanoparticles Loading Oxaliplatin as a Mucoadhesive Topical Treatment of Oral Tumors: Iontophoresis Further Enhances Drug Delivery Ex Vivo. Int. J. Biol. Macromol..

[29] Hassanpour M., Jafari H., Sharifi S., Rezaie J., Lighvan Z.M., Mahdavinia G.R., Gohari G., Akbari A., Hassanpour M., Jafari H. (2021). Salicylic Acid-Loaded Chitosan Nanoparticles (SA/CTS NPs) for Breast Cancer Targeting: Synthesis, Characterization and Controlled Release Kinetics. J. Mol. Struct..

[30] Liu Q., Tan Z., Zheng D., Qiu X. (2023). PH-Responsive Magnetic Fe_3_O_4_/Carboxymethyl Chitosan/Aminated Lignosulfonate Nanoparticles with Uniform Size for Targeted Drug Loading. Int. J. Biol. Macromol..

[31] Lohiya G., Katti D.S. (2022). Carboxylated Chitosan-Mediated Improved Efficacy of Mesoporous Silica Nanoparticle-Based Targeted Drug Delivery System for Breast Cancer Therapy. Carbohydr. Polym..

[32] Chen Q., Jia C., Xu Y., Jiang Z., Hu T., Li C., Cheng X. (2022). Dual-PH Responsive Chitosan Nanoparticles for Improving In Vivo Drugs Delivery and Chemoresistance in Breast Cancer. Carbohydr. Polym..

[33] Huang S.J., Wang T.H., Chou Y.H., Wang H.M.D., Hsu T.C., Yow J.l., Tzang B.S., Chiang W.H. (2022). Hybrid PEGylated Chitosan/PLGA Nanoparticles Designed as pH-Responsive Vehicles to Promote Intracellular Drug Delivery and Cancer Chemotherapy. Int. J. Biol. Macromol..

[34] Dhavale R.P., Dhavale R.P., Sahoo S.C., Kollu P., Jadhav S.U., Patil P.S., Dongale T.D., Chougale A.D., Patil P.B., Dhavale R.P. (2021). Chitosan Coated Magnetic Nanoparticles as Carriers of Anticancer Drug Telmisartan: pH-Responsive Controlled Drug Release and Cytotoxicity Studies. J. Phys. Chem. Solids.

[35] Kesavan S., Meena K.S., Sharmili S.A., Govindarajan M., Alharbi N.S., Kadaikunnan S., Khaled J.M., Alobaidi A.S., Alanzi K.F., Vaseeharan B. (2021). Ulvan Loaded Graphene Oxide Nanoparticle Fabricated with Chitosan and D-Mannose for Targeted Anticancer Drug Delivery. J. Drug Deliv. Sci. Technol..

[36] El-Shafai N.M., Masoud M.S., Ibrahim M.M., Ramadan M.S., Mersal G.A.M., El-Mehasseb I.M. (2022). Drug Delivery of Sofosbuvir Drug Capsulated with the β-Cyclodextrin Basket Loaded on Chitosan Nanoparticle Surface for Anti-Hepatitis C Virus (HCV). Int. J. Biol. Macromol..

[37] Kandile N.G., Mohamed H.M., Nasr A.S. (2020). Novel Hydrazinocurcumin Derivative Loaded Chitosan, ZnO, and Au Nanoparticles Formulations for Drug Release and Cell Cytotoxicity. Int. J. Biol. Macromol..

[38] Idoudi S., Hijji Y., Bedhiafi T., Korashy H.M., Uddin S., Merhi M., Dermime S., Billa N. (2022). A Novel Approach of Encapsulating Curcumin and Succinylated Derivative in Mannosylated-Chitosan Nanoparticles. Carbohydr. Polym..

[39] Rostami E. (2020). Progresses in Targeted Drug Delivery Systems Using Chitosan Nanoparticles in Cancer Therapy: A Mini-Review. J. Drug Deliv. Sci. Technol..

[40] Lee K.Y., Ha W.S., Park W.H. (1995). Blood Compatibility and Biodegradability of Partially N-Acylated Chitosan Derivatives. Biomaterials.

[41] Kamiyama K., Onishi H., Machida Y. (1999). Biodisposition Characteristics of N-Succinyl-Chitosan and Glycol-Chitosan in Normal and Tumor-Bearing Mice. Biol. Pharm. Bull..

[42] Barclay T.G., Day C.M., Petrovsky N., Garg S. (2019). Review of Polysaccharide Particle-Based Functional Drug Delivery. Carbohydr. Polym..

[43] Ahsan S.M., Thomas M., Reddy K.K., Sooraparaju S.G., Asthana A., Bhatnagar I. (2018). Chitosan as Biomaterial in Drug Delivery and Tissue Engineering. Int. J. Biol. Macromol..

[44] Lim C., Lee D.W., Israelachvili J.N., Jho Y., Hwang D.S. (2015). Contact Time- and pH-Dependent Adhesion and Cohesion of Low Molecular Weight Chitosan Coated Surfaces. Carbohydr. Polym..

[45] Shanmuganathan R., Edison T.N.J.I., LewisOscar F., Kumar P., Shanmugam S., Pugazhendhi A. (2019). Chitosan Nanopolymers: An Overview of Drug Delivery against Cancer. Int. J. Biol. Macromol..

[46] Felt O., Buri P., Gurny R. (1998). Chitosan: A Unique Polysaccharide for Drug Delivery. Drug. Dev. Ind. Pharm..

[47] Ahmed T., Aljaeid B. (2016). Preparation, Characterization, and Potential Application of Chitosan, Chitosan Derivatives, and Chitosan Metal Nanoparticles in Pharmaceutical Drug Delivery. Drug Des. Dev. Ther..

[48] Ładniak A., Jurak M., Wiącek A.E. (2021). Physicochemical characteristics of chitosan-TiO_2_ biomaterial. 2. Wettability and biocompatibility. Colloids Surf. A Physicochem. Eng. Asp..

[49] Ładniak A., Jurak M., Wiącek A.E. (2019). Langmuir Monolayer Study of Phospholipid DPPC on the Titanium Dioxide–Chitosan Hyaluronic Acid Subphases. Adsorption.

[50] Ładniak A., Jurak M., Wiącek A.E. (2022). The Effect of Chitosan/TiO_2_/Hyaluronic Acid Subphase on the Behaviour of 1,2-Dioleoyl-Sn-Glycero-3-Phosphocholine Membrane. Biomater. Adv..

[51] Mikušová V., Mikuš P. (2021). Advances in Chitosan-Based Nanoparticles for Drug Delivery. Int. J. Mol. Sci..

[52] Xie J., Lee S., Chen X. (2010). Nanoparticle-Based Theranostic Agents. Adv. Drug Deliv. Rev..

[53] Rampino A., Borgogna M., Blasi P., Bellich B., Cesàro A. (2013). Chitosan Nanoparticles: Preparation, Size Evolution and Stability. Int. J. Pharm..

[54] Rudramurthy G., Swamy M., Sinniah U., Ghasemzadeh A. (2016). Nanoparticles: Alternatives against Drug-Resistant Pathogenic Microbes. Molecules.

[55] Nagpal K., Singh S.K., Mishra D.N. (2010). Chitosan Nanoparticles: A Promising System in Novel Drug Delivery. Chem. Pharm. Bull..

[56] Zhang E., Xing R., Liu S., Qin Y., Li K., Li P. (2019). Advances in Chitosan-Based Nanoparticles for Oncotherapy. Carbohydr. Polym..

[57] Chen J., Guo Z., Tian H., Chen X. (2016). Production and Clinical Development of Nanoparticles for Gene Delivery. Mol. Ther. Methods Clin. Dev..

[58] Dmour I., Taha M.O. (2017). Novel Nanoparticles Based on Chitosan-Dicarboxylate Conjugates via Tandem Ionotropic/Covalent Crosslinking with Tripolyphosphate and Subsequent Evaluation as Drug Delivery Vehicles. Int. J. Pharm..

[59] Li Y., Zou Z., An J., Wu Q., Tong L., Mei X., Tian H., Wu C. (2022). Chitosan-Modified Hollow Manganese Dioxide Nanoparticles Loaded with Resveratrol for the Treatment of Spinal Cord Injury. Drug Deliv..

[60] Alonso M.J., Sánchez A. (2003). The Potential of Chitosan in Ocular Drug Delivery. J. Pharm. Pharmacol..

[61] Najafi S., Pazhouhnia Z., Ahmadi O., Berenjian A., Jafarizadeh-Malmiri H. (2014). Chitosan Nanoparticles and Their Applications in Drug Delivery: A Review. Curr. Res. Pharmacol. Drug Discov..

[62] Ali A., Ahmed S. (2018). A Review on Chitosan and Its Nanocomposites in Drug Delivery. Int. J. Biol. Macromol..

[63] Al-Musawi S., Jawad A., Hadi S.J., Hindi N.K. (2019). Preparation and Characterization of Folated Chitosan/Magnetic Nanocarrier for 5-Fluorouracil Drug Delivery and Studying Its Effect in Bladder Cancer Therap. J. Glob. Pharma Technol..

[64] Agnihotri S.A., Mallikarjuna N.N., Aminabhavi T.M. (2004). Recent Advances on Chitosan-Based Micro- and Nanoparticles in Drug Delivery. J. Control. Release.

[65] Garg U., Chauhan S., Nagaich U., Jain N. (2019). Current Advances in Chitosan Nanoparticles Based Drug Delivery and Targeting. Adv. Pharm. Bull..

[66] Sreekumar S., Goycoolea F.M., Moerschbacher B.M., Rivera-Rodriguez G.R. (2018). Parameters Influencing the Size of Chitosan-TPP Nano- and Microparticles. Sci. Rep..

[67] Yi Y., Wang Y., Liu H. (2003). Preparation of New Crosslinked Chitosan with Crown Ether and Their Adsorption for Silver Ion for Antibacterial Activities. Carbohydr. Polym..

[68] Yanat M., Schroën K. (2021). Preparation Methods and Applications of Chitosan Nanoparticles; with an Outlook toward Reinforcement of Biodegradable Packaging. React. Funct. Polym..

[69] Bellich B., D’Agostino I., Semeraro S., Gamini A., Cesàro A. (2016). “The Good, the Bad and the Ugly” of Chitosans. Mar. Drugs.

[70] Berger J., Reist M., Mayer J.M., Felt O., Peppas N.A., Gurny R. (2004). Structure and Interactions in Covalently and Ionically Crosslinked Chitosan Hydrogels for Biomedical Applications. Eur. J. Pharm. Biopharm..

[71] Bashir S.M., Ahmed Rather G., Patrício A., Haq Z., Sheikh A.A., Shah M.Z.l.H., Singh H., Khan A.A., Imtiyaz S., Ahmad S.B. (2022). Chitosan Nanoparticles: A Versatile Platform for Biomedical Applications. Materials.

[72] Mateescu M.A., Ispas-Szabo P., Assaad E. (2015). 3—Chitosan and Its Derivatives as Self-Assembled Systems for Drug Delivery. Controlled Drug Delivery.

[73] Shelma R., Paul W., Sharma C.P. (2010). Development and Characterization of Self-Aggregated Nanoparticles from Anacardoylated Chitosan as a Carrier for Insulin. Carbohydr. Polym..

[74] Quiñones J.P., Peniche H., Peniche C. (2018). Chitosan Based Self-Assembled Nanoparticles in Drug Delivery. Polymers.

[75] Des Rieux A., Fievez V., Garinot M., Schneider Y.J., Préat V. (2006). Nanoparticles as Potential Oral Delivery Systems of Proteins and Vaccines: A Mechanistic Approach. J. Control. Release.

[76] Zhao L., Duan X., Cao W., Ren X., Ren G., Liu P., Chen J. (2021). Effects of Different Drying Methods on the Characterization, Dissolution Rate and Antioxidant Activity of Ursolic Acid-Loaded Chitosan Nanoparticles. Foods.

[77] Saraswati L.D., Widjanarko B., Herawati V.E., Fauziah A.I. (2022). The Effects of Chitosan-PEG Nanoparticles Based on Channa Striata Protein Hydrolyzate on Decreasing Diabetes Mellitus in Diabetic Rats. Ethiop. J. Health Sci..

[78] Abdel-Moneim A., El-Shahawy A., Yousef A.I., Abd El-Twab S.M., Elden Z.E., Taha M. (2020). Novel Polydatin-Loaded Chitosan Nanoparticles for Safe and Efficient Type 2 Diabetes Therapy: In Silico, in Vitro and in Vivo Approaches. Int. J. Biol. Macromol..

[79] Mohanbhai S.J., Sardoiwala M.N., Gupta S., Shrimali N., Choudhury S.R., Sharma S.S., Guchhait P., Karmakar S. (2022). Colon Targeted Chitosan-Melatonin Nanotherapy for Preclinical Inflammatory Bowel Disease. J. Biomater. Adv..

[80] Coutinho A.J., Costa Lima S.A., Afonso C.M.M., Reis S. (2020). Mucoadhesive and PH Responsive Fucoidan-Chitosan Nanoparticles for the Oral Delivery of Methotrexate. Int. J. Biol. Macromol..

[81] Xavier F.H., Gueutin C., Chacun H., Vauthier C., Egito E.S.T. (2019). Mucoadhesive Paclitaxel-Loaded Chitosan-Poly (Isobutyl Cyanoacrylate) Core-Shell Nanocapsules Containing Copaiba Oil Designed for Oral Drug Delivery. J. Drug Deliv. Sci. Technol..

[82] Abdulmalek S., Mostafa N., Gomaa M., El-Kersh M., Elkady A.I., Balbaa M. (2022). Bee Venom-Loaded EGFR-Targeting Peptide-Coupled Chitosan Nanoparticles for Effective Therapy of Hepatocellular Carcinoma by Inhibiting EGFR-Mediated MEK/ERK Pathway. PLoS ONE.

[83] Sharma M., Sharma R., Jain D.K., Saraf A. (2019). Enhancement of Oral Bioavailability of Poorly Water Soluble Carvedilol by Chitosan Nanoparticles: Optimization and Pharmacokinetic Study. Int. J. Biol. Macromol..

[84] Lin C., Kuo T.C., Lin J.C., Ho Y.C., Mi F.L. (2020). Delivery of Polysaccharides from Ophiopogon Japonicus (OJPs) Using OJPs/Chitosan/Whey Protein Co-Assembled Nanoparticles to Treat Defective Intestinal Epithelial Tight Junction Barrier. Int. J. Biol. Macromol..

[85] Hassan N., Mirza M.A., Aslam M., Mahmood S., Iqbal Z. (2021). Doe Guided Chitosan Based Nano-Ophthalmic Preparation against Fungal Keratitis. Mater. Today Proc..

[86] Yu A., Shi H., Liu H., Bao Z., Dai M., Lin D., Lin D., Xu X., Li X., Wang Y. (2020). Mucoadhesive Dexamethasone-Glycol Chitosan Nanoparticles for Ophthalmic Drug Delivery. Int. J. Pharm..

[87] Rathore P., Mahor A., Jain S., Haque A., Kesharwani P. (2020). Formulation development, in vitro and in vivo evaluation of chitosan engineered nanoparticles for ocular delivery of insulin. RSC Adv..

[88] Ta Q., Ting J., Harwood S., Browning N., Simm A., Ross K., Olier I., Al-Kassas R. (2021). Chitosan Nanoparticles for Enhancing Drugs and Cosmetic Components Penetration through the Skin. Eur. J. Pharm. Sci..

[89] Abdel-Hafez S.M., Hathout R.M., Sammour O.A. (2018). Tracking the Transdermal Penetration Pathways of Optimized Curcumin-Loaded Chitosan Nanoparticles via Confocal Laser Scanning Microscopy. Int. J. Biol. Macromol..

[90] Rezaii M., Oryan S., Javeri A. (2019). Curcumin Nanoparticles Incorporated Collagen-Chitosan Scaffold Promotes Cutaneous Wound Healing through Regulation of TGF-Β1/Smad7 Gene Expression. Mater. Sci. Eng. C Mater. Biol. Appl..

[91] Truong T.H., Alcantara K.P., Bulatao B.P.I., Sorasitthiyanukarn F.N., Muangnoi C., Nalinratana N., Vajragupta O., Rojsitthisak P., Rojsitthisak P. (2022). Chitosan-Coated Nanostructured Lipid Carriers for Transdermal Delivery of Tetrahydrocurcumin for Breast Cancer Therapy. Carbohydr. Polym..

[92] Talib S., Ahmed N., Khan D., Khan G.M., Rehman A. (2021). Chitosan-Chondroitin Based Artemether Loaded Nanoparticles for Transdermal Drug Delivery System. J. Drug Deliv. Sci. Technol..

[93] Tolentino S., Pereira M.N., Cunha-Filho M., Gratieri T., Gelfuso G.M. (2021). Targeted Clindamycin Delivery to Pilosebaceous Units by Chitosan or Hyaluronic Acid Nanoparticles for Improved Topical Treatment of Acne Vulgaris. Carbohydr. Polym..

[94] Takeuchi I., Takeshita T., Suzuki T., Makino K. (2017). Iontophoretic Transdermal Delivery Using Chitosan-Coated PLGA Nanoparticles for Positively Charged Drugs. Colloids Surf. B Biointerfaces.

[95] Friedman A.J., Phan J., Schairer D.O., Champer J., Qin M., Pirouz A., Blecher-Paz K., Oren A., Liu P.T., Modlin R.L. (2013). Antimicrobial and Anti-Inflammatory Activity of Chitosan-Alginate Nanoparticles: A Targeted Therapy for Cutaneous Pathogens. J. Investig. Dermatol..

[96] Lopes Rocha Correa V., Assis Martins J., Ribeiro de Souza T., de Castro Nunes Rincon G., Pacheco Miguel M., Borges de Menezes L., Correa Amaral A. (2020). Melatonin Loaded Lecithin-Chitosan Nanoparticles Improved the Wound Healing in Diabetic Rats. Int. J. Biol. Macromol..

[97] Choudhary A., Kant V., Jangir B.L., Joshi V.G. (2020). Quercetin Loaded Chitosan Tripolyphosphate Nanoparticles Accelerated Cutaneous Wound Healing in Wistar Rats. Eur. J. Pharmacol..

[98] Teixeira-Santos R., Lima M., Gomes L.C., Mergulhão F.J. (2021). Antimicrobial Coatings Based on Chitosan to Prevent Implant-Associated Infections: A Systematic Review. iScience.

[99] Yilmaz Atay H. (2020). Antibacterial Activity of Chitosan-Based Systems. Functional Chitosan.

